# The investigation of precipitation behavior of titanium compounds for high titanium steel based on in situ observation

**DOI:** 10.1371/journal.pone.0275049

**Published:** 2023-04-03

**Authors:** Xiaolei Zhu, Ji Yang, Shuang Wang, Shenchuan Bu, Minggang Shen, Xincheng Miao, Xiang Li

**Affiliations:** 1 School of Materials and Metallurgy, University of Science and Technology, Anshan, China; 2 Iron & Steel Research Institutes of Ansteel Group Corporation, Anshan, China; Pacific Northwest National Laboratory, UNITED STATES

## Abstract

The effects of cooling rate, Ti content, and casting temperature on titanium compounds for high titanium steel were investigated. In-situ observation of high titanium steel during remelting and solidification was carried out by using a High Temperature Confocal Scanning Laser Microscope (HTCSLM), and the observed results were in good agreement with the thermodynamic and kinetic calculations. The observation and calculation results both show that the inclusions in high titanium steel first precipitate in the form of TiN, followed by TiC precipitates as temperature decreases, eventually forming TiCxN1-x type inclusions at room temperature. The initial precipitation temperature of the inclusions increases with the increase of Ti content in molten steel, whereas casting temperature has little effect on the initial precipitation temperature of inclusions. In addition, the size of TiN inclusions increases with the increase of Ti content in steel but decreases with the increase in cooling rate.

## 1. Introduction

Compared with Nb and V, Ti is more abundant in resources and cheaper in price, which has attracted more and more attention in recent years [1, 2]. At present, researchers try to replace some expensive Nb with cheap Ti for microalloying, and adopt a high-purity smelting process and controlled rolling and controlled cooling process to achieve grain refinement and precipitation strengthening, to obtain high-strength and toughness steel materials [3]. However, for the smelting process of high titanium steel, Ti is easy to react with the N and C in the steel to precipitate the inclusions of TiN and TiC. However, in the smelting process, Ti is easy to react with the N to precipitate the inclusion of TiN in the steel, which reduces the effective titanium content. In addition, the precipitation of large-size TiN inclusions will be harmful to the material performance, and will also adhere to the inside of the submerged entry nozzle of continuous casting, resulting in the nozzle clogging [4].

The inclusion of TiC in high titanium steel has the characteristics of low density and high hardness, which can be used to improve the wear resistance of materials [5, 6]. In addition, if Ti and N in high titanium steel form stable titanium compound TiN during the solidification process, it can hinder the migration of austenite grain boundary, further refine austenite grain and improve the strength and toughness of the steel [7, 8]. TiC precipitates in the form of a eutectic reaction in high Ti steel, so the size of the precipitate is large. Eutectic TiC has the characteristics of low density and high hardness, so it can be used to improve the wear resistance of materials [9, 10]. The toughness and plasticity of high titanium wear-resistant steel do not decrease but increase with the increase of strength, which is mainly due to the refinement and homogenization of large-size TiC during rolling deformation [11]. Yang et al. [12] found that the inclusions of TiN were square with sharp edges, which were not easy to deform, but would damage the toughness of steel. A similar conclusion was also proposed by Fu et al. [13]. Many researchers [14–16] had found that the precipitation and growth of TiN inclusions in steel could be controlled by reducing the content of Ti and N. Huo et al. [17] studied the behavior of precipitates in steels with different Ti contents and found that the size of TiN increased with the increase of Ti content. Because TiN and TiC had the same crystal structure of NaCl type, and the lattice constant was almost the same, the two inclusions usually completely dissolved each other to form Ti(NC) [18]. Cheng et al. [19] proposed that TiN would precipitate in the liquid phase, and its size was related to the content of Ti and N in the molten steel. Besides, the cooling rate was also an influencing factor for precipitating TiN. However, the precipitation behavior of titanium compounds is not only related to the cooling rate and element composition but also related to the casting process of continuous casting. At present, there is still a lack of systematic research on the precipitation behavior of TiN or TiC related to the continuous casting process.

In this work, the HTCSLM experiment was used to observe the solidification process of the mushy zone for high titanium steel and the precipitation behavior of titanium compounds. The effects of Ti content, cooling rate, and casting temperature on the precipitation behavior of titanium compound high titanium steel were investigated systematically. The thermodynamic and dynamic calculations of titanium compound growth were carried out simultaneously. The influence of different Ti content, different cooling rate, and casting temperatures on the precipitation behavior of titanium inclusion in steel was investigated by using HTCSLM and thermodynamic kinetic theory. Furthermore, the experimental results were in agreement with the calculated results.

## 2. Experiment design and method

### 2.1 Material

**Fig 1:**
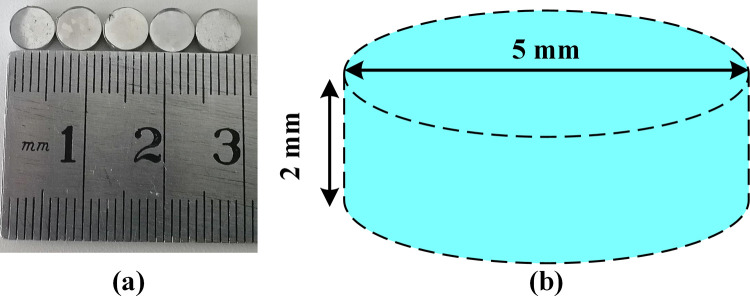
The samples for the HTCSLM experiment and their detailed dimensions.

In this experiment, the high titanium steel was melted by a 200 kg vacuum induction furnace. The unit for supplying this material is the Iron & Steel Research Institutes of Ansteel Group Corporation. The intervention of a vacuum chamber can greatly avoid oxidation reactions in the smelting process. Three kinds of high titanium steels with different titanium contents were prepared simultaneously to investigate the effect of titanium elements on solidification characteristics. Here, Fe-0.3wt.%Ti, Fe-0.5wt.%Ti, and Fe-0.7wt.%Ti were used to distinguish three kinds of high titanium steels with different titanium contents. The chemical compositions of the target steel are shown in Table 1. Besides, the HTCSLM experiment was carried out in this work. The samples for the HTCSLM experiment and their detailed dimensions are shown in Fig 1. The samples are cylindrical, the diameter of sample is 5mm and the height is 2mm.

**Table 1:** The main chemical compositions of the high titanium steel.

Target steel	Components, wt.%									
	C	Si	Mn	P	S	Al	Ni	Ti	Mo	N
Fe-0.3wt.%Ti	0.40	1.21	3.52	0.008	0.0052	0.022	1.71	0.31	0.15	0.0043
Fe-0.5wt.%Ti	0.41	1.22	3.51	0.011	0.0054	0.020	1.73	0.50	0.14	0.0045
Fe-0.7wt.%Ti	0.41	1.21	3.54	0.009	0.0047	0.022	1.71	0.71	0.15	0.0042

### 2.2 The experimental method

The laser scanning confocal microscope (LSCM) was used to observe the solidification process of high titanium steel. The model of HTCSLM is VL2000DX, and its manufacturer is YONEKURA MFG CO., LTD. The effects of cooling rate, titanium content in steel, and casting temperature on the precipitation behavior of titanium compounds were studied by a single factor experiment. In this experiment, the detailed cooling rate, titanium content in steel, and casting temperature are shown in Table 2.

**Table 2:** The main chemical compositions of the high titanium steel.

	Cooling rate, K/min	Titanium content, wt.%	Casting temperature, K
Group 1	282	0.5wt.%Ti	1773
	285		
	288		
	291		
	294		
Group 2	288	0.3wt.%Ti	1773
		0.5wt.%Ti	
		0.7wt.%Ti	
Group 3	288	0.5wt.%Ti	1773
			1793
			1813

The detailed experimental process is as follows:

First, the sample (0.5Ti) was heated from room temperature to 473 K at a heating rate of 313 K/min. Then, the sample was heated to 1723 K at a heating rate of 873K/min. Between 1723 K and 1773 K, the sample was heated at a heating rate of 473K/min. Keep constant temperature for 0.5 min at 1773 K to ensure complete melting of the sample; Next, the molten samples were cooled to 1573 K at the cooling rate of 282, 285, 288, 291, and 294 K/min respectively, and finally, the samples were cooled from 1573K to room temperature at the cooling rate of 873K/min. During the heating, melting and cooling, and solidification process, the phase transformation behavior was recorded at the rate of 15 frames/sec. All experiments were performed by Table 2.

## 3 Experimental results

### 3.1 In situ observation of high titanium steel during remelting and solidification

**Fig 2:**
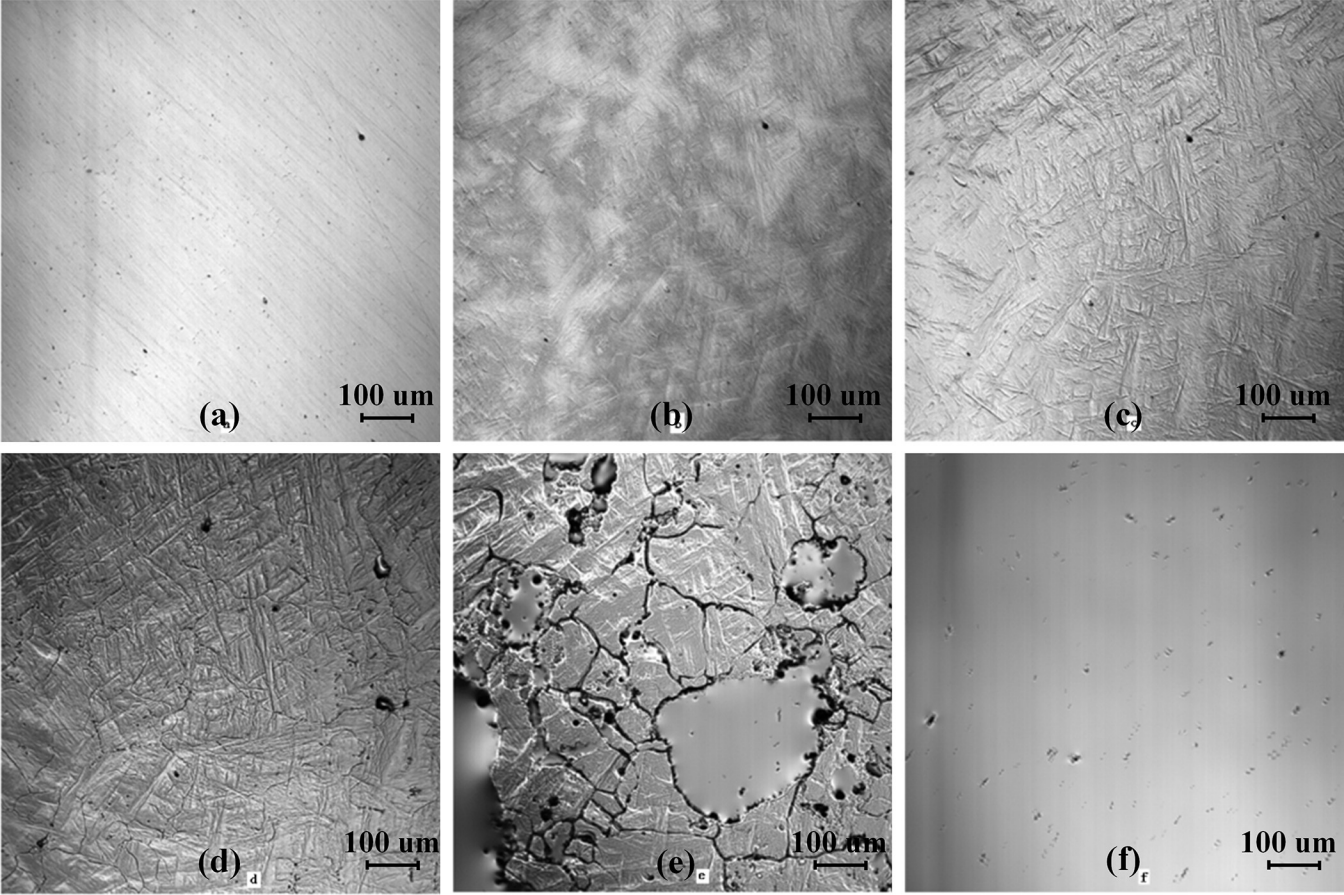
The remelting process of high titanium steel (Fe-0.5wt.%Ti) at a cooling rate of 288 K/min, (a) 293K; (b) 1041K; (c) 16455K; (d) 1703K; (e) 1734K; (f) 1751K.

The remelting process of the high titanium steel (Fe-0.5wt.%) at a cooling rate of 288 K/min is shown in Fig 2. Fig 2(A) shows the surface morphology of high titanium steel at room temperature. When the temperature increases, grain boundaries gradually appear, as shown in Fig 2(B). When the heating process is continued, the grain boundary becomes more and more clear and appears completely. At the same time, the grain grows gradually and the color is deeper, as shown in Fig 2(C) and 2(D). However, when the temperature reaches 1713 K, the grain boundary melts first, and then the grain inside begins to melt. The liquid phase appears on the surface of the sample, as shown in Fig 2(E). When the temperature reaches 1751 K, the sample has completely melted, as shown in Fig 2(F).

**Fig 3:**
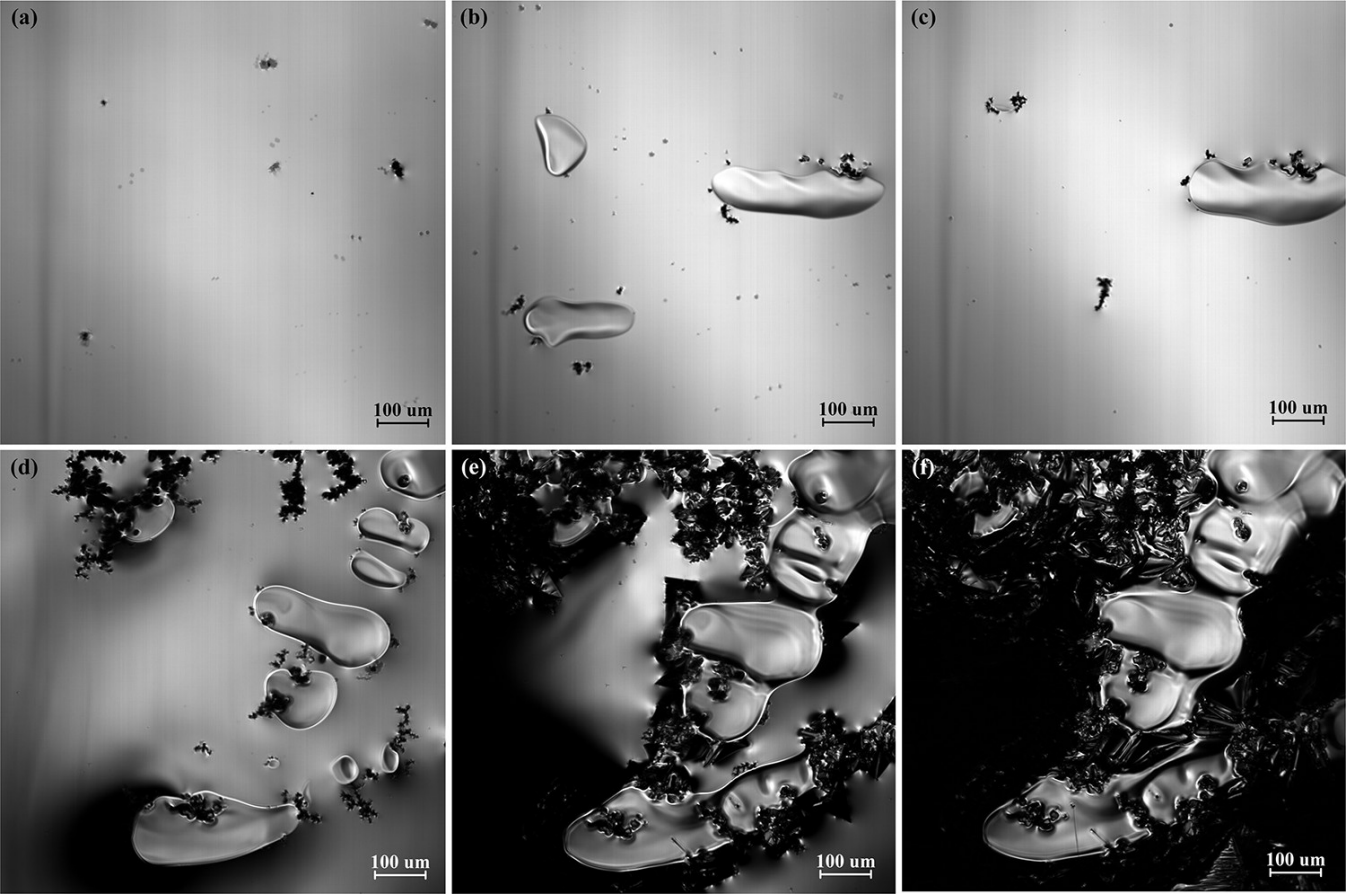
The solidification process of high titanium steel (Fe-0.5wt.%Ti) at a cooling rate of 288 K/min, (a) 1763K; (b) 1693K; (c) 1682K; (d) 1671K; (e) 1660K; (f) 1644K.

Fig 3 shows the solidification process of the high titanium steel (Fe-0.5wt.%) at a cooling rate of 288 K/min. Before solidification, some black TiN inclusions of micron-scale float on the surface of molten steel, as shown in Fig 3(A). As the temperature decreases, TiN inclusions continue to precipitate, the contact increases and the aggregation becomes larger and coarser, as shown in Fig 3(B)–3(D). When the temperature decreases to 1649 K (in the mushy zone), TiC inclusions begin to precipitate. Compared with TiN, the size of TiC inclusions is larger, so the newly precipitated TiC inclusions will continuously gather with TiN inclusions, forming TiC_*x*_N_1-*x*_ inclusions, as shown in Fig 3(E) and 3(F).

**Fig 4:**
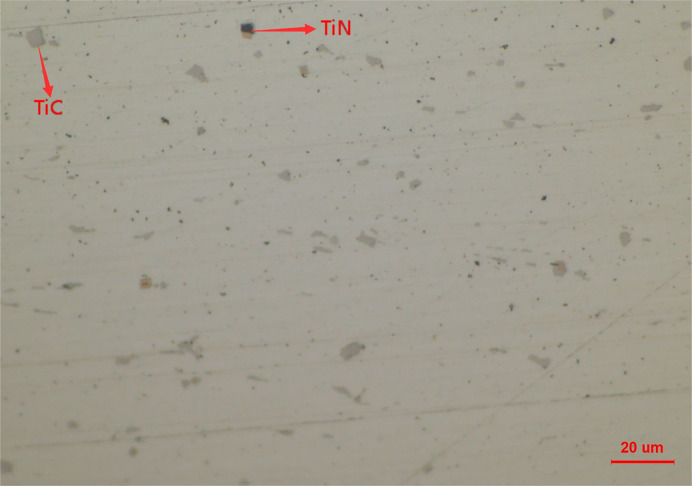
The titanium compounds in high titanium steel (Fe-0.5wt.%Ti) at room temperature (298 K).

As the sample of high titanium steel is cooled to room temperature, the color and contrast of the precipitated TiN and TiC inclusions under the metallurgical microscope are also different, as shown in Fig 4. It is confirmed from Fig 3 that the size of TiC inclusions is larger than that of TiN inclusions. With the decrease in temperature, TiC inclusions precipitate from the liquid phase and TiN phase and then wrap the TiN inclusions. Finally, TiC_*x*_N_1-*x*_ inclusions are formed at room temperature.

### 3.2 Effect of different cooling rates on precipitation of titanium compounds

**Fig 5:**
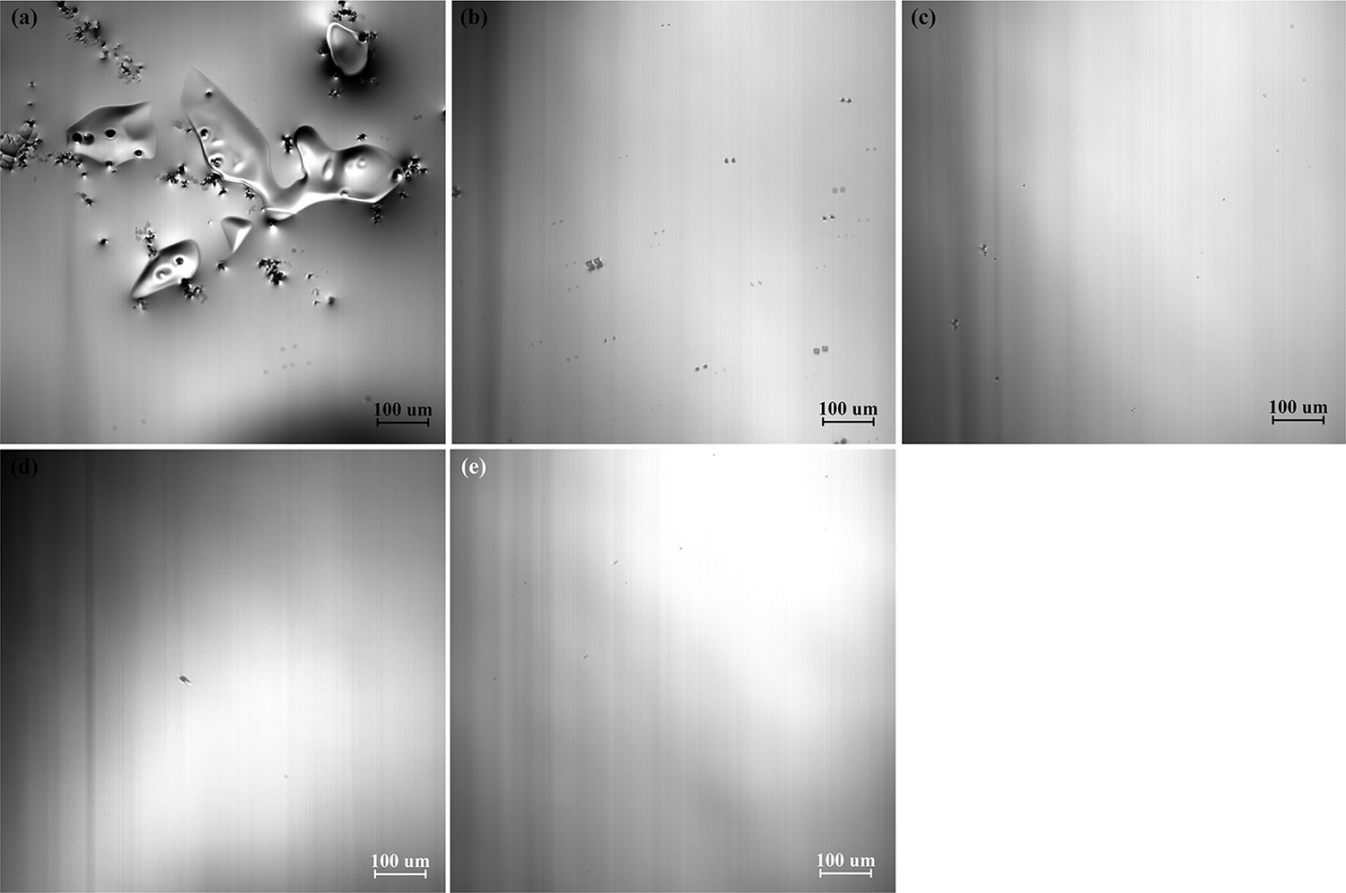
The precipitation of titanium compounds (Fe-0.5wt.%Ti) and the image temperature is 1703K at different cooling rates of (a) 282 K/min, (b) 285 K/min, (c) 288 K/min, (d) 291 K/min, and (e) 294 K/min.

Fig 5 shows the titanium compounds precipitation of the high titanium steel (Fe-0.5wt.%) at different cooling rates. The precipitation of titanium compounds has a relationship with the increase in cooling rate at 1707 K. When the cooling rate is 282 K/min, TiN precipitated from the liquid phase and grew up at 1707 K, as shown in Fig 5(A). As the cooling rate gradually increases, the TiN precipitated in the liquid phase is more refined and the precipitation amount gradually decreases, as shown in Fig 5(B)–5(E). The above analysis shows that the cooling rate of molten steel affects the precipitation amount and precipitation time of titanium compounds, and increasing the cooling rate of molten steel can delay the precipitation of titanium compounds in the liquid phase to a certain extent.

### 3.3 Effect of different titanium contents on precipitation of titanium compounds

**Fig 6:**
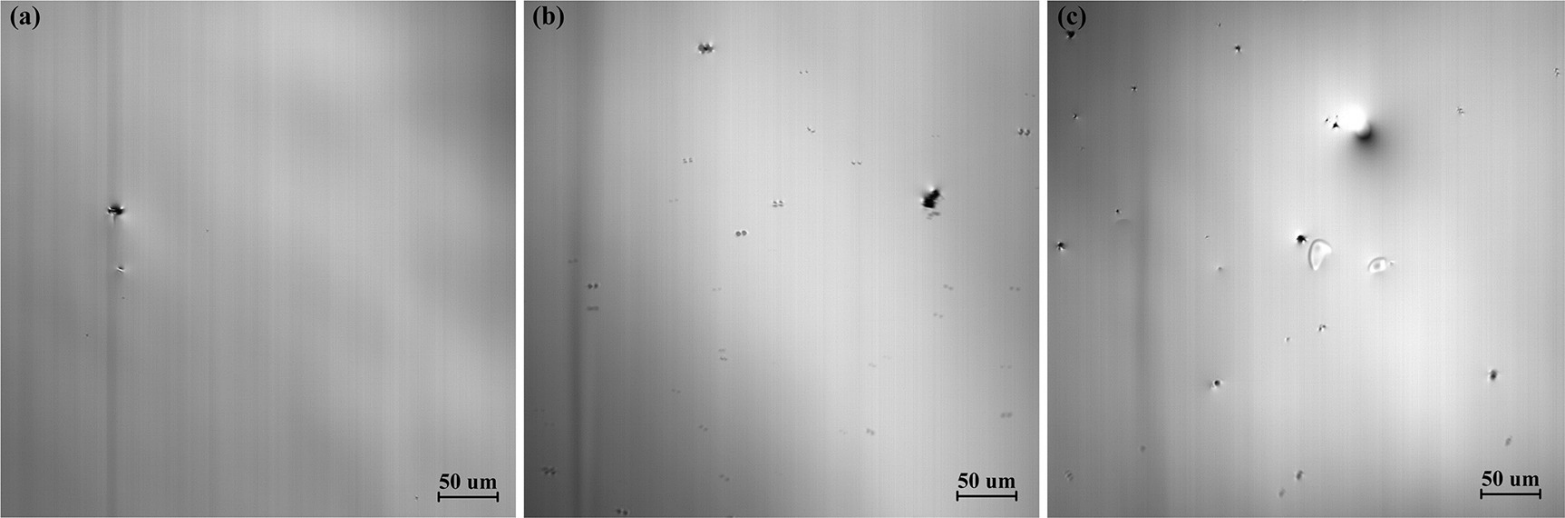
The precipitation of titanium compounds at different titanium contents of (a) 0.3 wt.% Ti, (b) 0.5 wt.% Ti, and (c) 0.7 wt.% Ti, and the image temperature is 1683K at cooling rate of 288 K/min.

As shown in Fig 6, HTCSLM recorded the precipitation of titanium compounds with different titanium contents of (a) 0.3 wt.% Ti, (b) 0.5 wt.% Ti, and (c) 0.7 wt.% Ti, respectively. Increasing the titanium content in the high titanium steel has a certain effect on the precipitation of titanium compounds in the liquid phase. At 1738 K, when the Ti content in the sample is 0.3 wt.%, a small number of titanium precipitates were observed, as shown in Fig 6(A). When the Ti content in the steel increases to 0.5 wt.%, the number of titanium compounds that can be precipitated at 1738K increases, as shown in Fig 6(B) and 6(C) is an in-situ observation image of titanium compounds precipitated in the liquid phase with a titanium content of 0.7 wt.% Ti at 1738 K. Compared with a sample with a titanium content of 0.3 wt.% Ti and 0.5 wt.% Ti, the titanium compounds precipitated in the liquid phase are further refined and the amount is significantly increased for the sample with a titanium content of 0.7 wt.% Ti. Generally, with the increase of titanium content, the precipitation temperature of titanium compounds from the liquid phase increases, and the precipitation amount of titanium compounds also increase.

### 3.4 Effect of different casting temperatures on precipitation of titanium compounds

**Fig 7:**
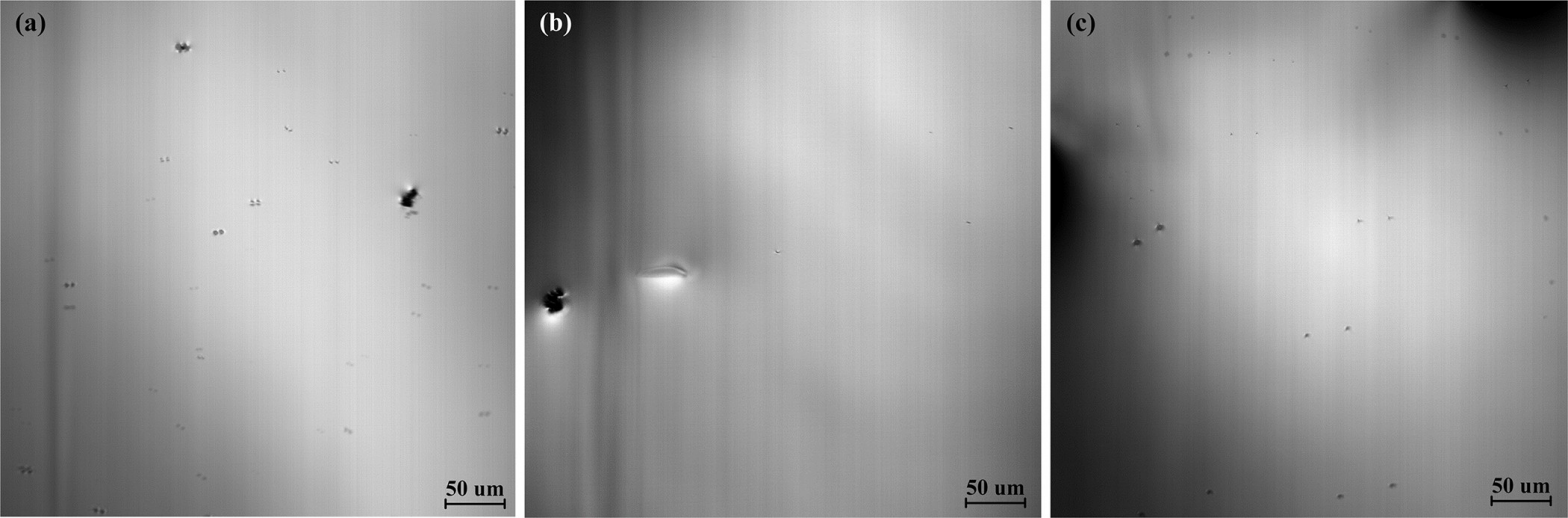
The precipitation of titanium compounds (Fe-0.5wt.%Ti) at different casting temperatures of (a) 1773 K, (b) 1793 K, and (c) 1813 K and the image temperature is 1673K at cooling rate of 288 K/min.

Fig 7 shows the precipitation of titanium compounds of the high titanium steel (Fe-0.5wt.%) at room temperature with different casting temperatures (1773 K, 1793 K, and 1813K). The casting temperature has little effect on the precipitation of titanium compounds in the molten steel, and the casting temperature has little effect on the precipitation temperature, number, and size of titanium compounds.

## 4 Analysis and discussion

### 4.1 The calculation of liquidus and solidus temperatures for high titanium steel

According to the chemical composition in high titanium steel, the liquidus and solidus temperatures were calculated according to Eq (1) [19].

$$T_{l}=1811‐\sum \Delta T\cdot w[i]$$ (1)

$$T_{s}=1811‐\sum \Delta T\cdot w[i]$$ (2)

where *T*_*ℓ*_ and *T*_s_ are the liquidus and solidus temperatures, *w*[*i*] representing the element contents. In Eq (1), Δ*T* is the effects of element contents on the temperature, which is shown in Table 3 [19]. For the high titanium steel, the liquidus and solidus temperatures can be obtained based on the steel components in Table 1, which is shown in Table 4.

**Table 3:** The effect of element content on the liquidus and solidus temperatures.

Components	C	Si	Mn	P	S	Al	Ni	Ti	Mo	N
Δ*T* (liquidus temperature), K	65	8	5	30	25	3	4	20	2	90
Δ*T* (solidus temperature), K	175	20	30	280	575	7.5	4.75	40	5	/

**Table 4:** The liquidus and solidus temperatures for the high titanium steel.

Target steel	Titanium content, wt.%	liquidus temperature, K	solidus temperature, K
Fe-0.3wt.%Ti	0.3wt.%Ti	1746	1594
Fe-0.5wt.%Ti	0.5wt.%Ti	1742	1586
Fe-0.7wt.%Ti	0.7wt.%Ti	1738	1578

### 4.2 Theoretical analysis of TiN precipitation

The thermodynamic equilibrium condition for TiN formation in high titanium steel are as follows:

$$[\mathrm{Ti}]+[N]={\mathrm{TiN}}_{\left(s\right)}\;\Delta G^{\theta }=‐291000+107.91\cdot T$$ (3)

$$\Delta G=\Delta G^{\theta }+RT\ln\frac{1}{\left(w[\mathrm{Ti}]\cdot f_{\mathrm{Ti}})\cdot (w[N]\cdot f_{N}\right)}$$ (4)

where *w*[Ti] and *w*[N] are the contents of nitrogen and titanium in molten steel respectively, *f*_Ti_ and *f*_N_ are the activity coefficients of nitrogen and titanium.

The relationship between the activity coefficients of Ti and N and the content of each element in steel is as follows:

$$\mathrm{lg}f_{i}=\sum e_{i}^{j}\times w[j]$$ (5)

When the temperature is 1873 K, the steel is fully molten, and the interaction coefficient of each element is shown in Table 5 [20].

**Table 5:** The interaction coefficient of each element (1873 K).

Element (*i*)	Element (*j*)									
	C	Si	Mn	P	S	Al	Ni	Ti	Mo	N
Ti	-0.1650	0.0500	0.0043	-0.0064	-0.1100	/	/	0.0130	/	-1.8000
N	0.1300	0.0470	-0.0210	0.0450	0.0070	-0.0280	0.0100	-0.5300	-0.0110	0
C	0.1400	0.0800	-0.0120	0.0510	0.0460	0.0430	0.0120	/	-0.0083	0.1100

The relationship between the activity coefficients of Ti and N in molten steel and temperature (*T*) is as follows:

$$\mathrm{lg}f_{\mathrm{Ti},T}=(2557/T-0.365)\mathrm{lg}f_{\mathrm{Ti},1873K}$$ (6)

$$\mathrm{lg}f_{N,T}=(3280/T-0.70)\mathrm{lg}f_{N,1873K}$$ (7)

For three target steels (Fe-0.3wt.%Ti, Fe-0.5wt.%Ti, and Fe-0.7wt.%Ti), the titanium element and nitrogen element can have three combinations: *w*[Ti] = 0.3 wt.% and *w*[N] = 0.0045 wt.%, *w*[Ti] = 0.5 wt.% and *w*[N] = 0.0045 wt.%, *w*[Ti] = 0.7 wt.% and *w*[N] = 0.0045 wt.%. The following information can be obtained by substituting these three combinations with different element contents into the Eqs (3) ~ (7) and Table 4. When ΔG is equal to 0, the initial precipitation temperature of TiN is 1763 K, 1785 K, and 1792 K, which are higher than the liquidus temperature. It shows that TiN can be precipitated in the liquid phase when *w*[Ti] is greater than 0.3 wt.%, so a few inclusions can be observed floating in Fig 2(A). The change of Ti content has a direct effect on the initial precipitation temperature of TiN and is in direct proportion. With the increase of Ti content in the steel, the precipitation temperature of TiN increases, that is, the precipitation amount of titanium compound for Fe-0.7wt.%Ti is the largest.

When the Ti content in the steel is greater than 0.3 wt.%, TiN has begun to form in the liquid phase, and the precipitation temperature of TiN increases with the increase of the Ti content in the steel. During solidification, the titanium and nitrogen elements in the mushy zone will be segregated and enriched. Besides, the decrease in temperature changes the equilibrium concentration product (*K*_TiN_) of TiN, so the actual activity product (*Q*_TiN_) of TiN is much larger than *K*_TiN_, and TiN will always precipitate, aggregate, and grow.

### 4.3 Theoretical analysis of TiC precipitation

The thermodynamic equilibrium condition for forming TiC in steel are as follows:

$$[\mathrm{Ti}]+[C]=\mathrm{TiC}(s)\;\Delta G^{\theta }=‐182290+99.79T$$ (8)

$$\Delta G=\Delta G^{\theta }+RT\ln\frac{a_{\mathrm{TiC}}}{a_{\mathrm{Ti}}\cdot a_{C}}=\Delta G^{\theta }‐RT\ln[\left(w[\mathrm{Ti}]\cdot f_{\mathrm{Ti}})\cdot (w[C]\cdot f_{C}\right)]$$ (9)

where *w*[Ti] and *w*[C] are the contents of titanium and carbon, and *f*_Ti_ and *f*_C_ are the activity coefficients of titanium and carbon, respectively.

For three target steels (Fe-0.3wt.%Ti, Fe-0.5wt.%Ti, and Fe-0.7wt.%Ti), the titanium element and carbon element can have three combinations: *w*[Ti] = 0.3 wt.% and *w*[C] = 0.4 wt.%, *w*[Ti] = 0.5 wt.% and *w*[C] = 0.4 wt.%, *w*[Ti] = 0.7 wt.% and *w*[C] = 0.4 wt.%. The following information can be obtained by substituting three combinations with different element contents into the above equations. When ΔG is equal to 0, the initial precipitation temperature of TiC is 1589 K, 1651 K, and 1695 K, which are lower than the liquidus temperature. It shows that TiC can be precipitated in the solid phase when *w*[Ti] is greater than 0.3 wt.%. With the increase of Ti content in the steel, the precipitation temperature of TiC also increases. When *w*[Ti] reaches 0.5 wt.% or 0.7 wt.%, TiC will precipitate in the mushy zone.

During solidification, the actual activity product (*Q*_TiC_) of Ti and C at the front of the solidification interface is expressed as:

$$Q_{\mathrm{TiC}}=f_{\mathrm{Ti}}\cdot f_{C}\cdot w[\mathrm{Ti}]\cdot w[C]$$ (10)

where *w*[Ti] and *w*[C] are the actual contents of titanium and carbon at the front of the solidification interface.

The expression of the actual contents of titanium at the front of the solidification interface is as follows.

$$w[\mathrm{Ti}]=\frac{w{\left[\mathrm{Ti}\right]}_{0}}{1‐(1‐k_{\mathrm{Ti}})f_{s}}$$ (11)

where *w*[Ti]_0_ is the initial mass fraction of titanium in molten steel, and *k*_Ti_ is the equilibrium distribution coefficient of titanium in the mushy zone, which is equal to 0.32 in this work. *f*s is the solid phase fraction.

The expression of the actual contents of carbon at the front of the solidification interface is as follows.

$$w[C]=\frac{w{\left[C\right]}_{0}}{1‐(1‐k_{C})f_{s}}$$ (12)

where *w*[C]_0_ is the initial mass fraction of carbon in molten steel, and *k*_C_ is the equilibrium distribution coefficient of carbon in the mushy zone, which is equal to 0.17 in this work.

The changing trend of the equilibrium constant (*K*_TiC_) for precipitating TiC with temperature can be calculated by the following equation:

$$\mathrm{lg}K_{\mathrm{TiC}}=5.317‐\frac{9393}{T}$$ (13)

The temperature (*T*) at the front of the solidification interface can be calculated by the following equation:

$$T=T_{\mathrm{Fe}}‐\frac{T_{\mathrm{Fe}}‐T_{l}}{1‐f_{s}\frac{T_{l}‐T_{s}}{T_{\mathrm{Fe}}‐T_{s}}}$$ (14)

where *T* is the temperature, and *T*_Fe_ is the melting temperature of pure iron (1809 K).

**Fig 8:**
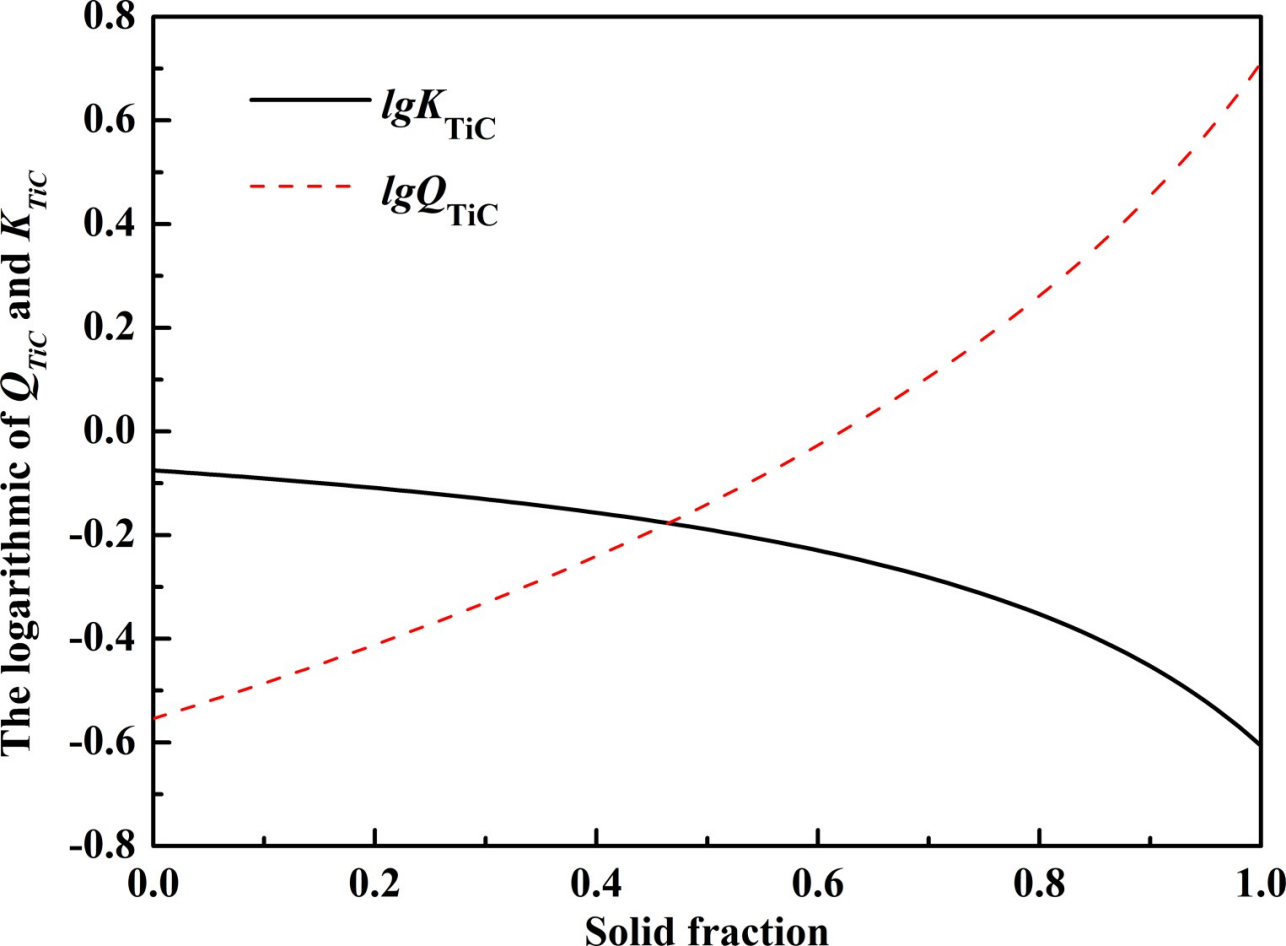
The relationship between lg*Q*_TiC_, lg*K*_TiC_, and solid fraction.

When *w*[Ti] is equal to 0.5 and *w*[C] equal to 0.4, the relationship between lg*Q*_TiC_, lg*K*_TiC_, and *f*_S_ can be calculated, which is shown in Fig 8. In the solidification process, the actual activity product (*Q*_TiC_) of TiC is larger than the equilibrium activity product (*K*_TiC_). It can prove that TiC will precipitate from the mushy zone. It is not difficult to find from Fig 8 that with the continuous reduction of temperature, the equilibrium activity product (*K*_TiC_) becomes smaller and smaller. This is because of Ti and C in the steel form TiC inclusions and precipitate. At the end of solidification, the reaction becomes more intense, and more and more titanium inclusions will gather. This phenomenon can also be verified from the results of in situ observation of high titanium steel.

The thermodynamic calculation shows that TiN inclusions have been formed and precipitated in the molten steel of high titanium steel before solidification. In the solidification process, the reaction to form TiN will still be violent, and the TiN inclusions will further precipitate and aggregate with each other. With the continuous decrease of temperature, TiC inclusions begin to form in molten steel, and more TiC inclusions are formed under the effect of the gradual enrichment of elements. The newly precipitated TiC inclusions and TiN inclusions aggregate and grow together, and finally form TiC_*x*_N_1-*x*_ inclusions under the growth conditions of a solid solution, which is consistent with the in-situ observation results. In addition, the precipitation temperatures of TiN and TiC are related to the content of Ti in molten steel and have nothing to do with the casting temperature. The number and size of TiN and TiC are also independent of casting temperature. Therefore, changing the casting temperature does not affect the precipitation temperatures of TiN and TiC.

Since the Ti content of the new high titanium steel is much higher than that of the conventional titanium micro-alloyed steel, the Ti in the molten steel will form TiC_*x*_N_1-*x*_ inclusions which precipitated with N and C. The precipitates can pin the austenite grain boundary and hinder the austenite grain growth. The plate-like martensite formed after quenching will also become fine, which is conducive to improving the wear resistance of high titanium steel. The improvement of wear resistance mainly comes from the blocking effect of micron TiC inclusions on the “furrow”, and the wear resistance (*ɛ*) is approximately linear to the mass fraction of TiC inclusions in steel, which is shown as follows [21, 22]:

ε=1+w[TiC] (15)

Here, *w*[TiC] is the mass fraction of TiC.

It can be seen that the presence of TiC inclusions can significantly improve the wear resistance of steel without increasing the hardness. When the Ti content increases from 0.3 wt.% to 0.5 wt.% or even 0.7 wt.%, the precipitation amount of TiN and TiC in the micron and nanoscale increases obviously, which significantly improves the wear resistance of the high titanium steel. However, due to the extremely high hardness of micron TiC (3200 HV), the incongruous deformation at the interface between the precipitated phase and matrix can easily induce cracks in the matrix; At the same time, the mosaic stress caused by the thermal expansion difference between the precipitated phase and the matrix also easily promotes the fracture of TiC itself, and the matrix fracture will be induced after the crack further extends to the matrix. Therefore, TiC particles of micron size will lead to a decrease in toughness and plasticity for the high titanium steel [23, 24]. Too low Ti content can not help to improve the wear resistance of steel, while too high Ti content will reduce the toughness and plasticity of steel. Therefore, a reasonable Ti content should be formulated in combination with hot rolling and heat treatment processes to improve the mechanical properties of the steel and adapt to the application environment of the product to the greatest extent.

### 4.4 Effect of Ti content and cooling rate on the TiN size

According to the previous thermodynamic calculation, TiN has been formed and accumulated in the liquid phase. With the progress of the solidification process of molten steel, TiN inclusions continue to accumulate at the front of the solidification interface, and TiN inclusions continue to precipitate and grow with the decrease in temperature. The growth behavior of precipitated TiN in the liquid phase determines its final size. The content of nitrogen in high titanium steel is much lower than that of titanium. Therefore, the final size of TiN is affected by different content of titanium and the change in cooling rate.

The diffusion of titanium in steel is slower than that of nitrogen, and it is easier to enrich at the front of the solidification interface. Because of this, the diffusion of nitrogen in the liquid phase has traditionally been regarded as the limiting factor for the growth of TiN particles. Therefore, with the advancement of the solidification process of molten steel, the kinetic equation of TiN inclusion growth in the solidification process of high titanium steel is as follows [25].


$$r\frac{dr}{dt}=\frac{M_{\mathrm{TiN}}\rho_{\mathrm{Fe}}}{M_{\mathrm{Fe}}\rho_{\mathrm{TiN}}}D_{N}\left\{w[N\right]‐w{\left[N\right]}_{\mathrm{eq}}\}$$ (16)

By integrating the Eq (16), the following equation can be obtained.

$$r=10^{4}\sqrt{\frac{M_{\mathrm{TiN}}}{50M_{\mathrm{Fe}}}\cdot \frac{\rho_{\mathrm{Fe}}}{\rho_{\mathrm{TiN}}}D_{N}\{w[N]‐w{\left[N\right]}_{\mathrm{eq}}\}\tau }$$ (17)

where *r* is the radius of TiN inclusion, *t* is the solidification time, *τ* is the local solidification time which is related to the cooling rate, *w*[N] is the mass fraction of N in molten steel, *w*[N]_eq_ is the equilibrium mass fraction of N, *D*_N_ is the diffusion coefficient of N in molten steel which is equal to [3.25×10^−3^ exp(-11500/RT)], M_TiN_ is the relative molecular mass of TiN (62 g/mol), M_Fe_ is the relative molecular mass of Fe (56 g/mol), *ρ*_Fe_ is the steel density (7.07 g/cm^3^), and *ρ*_TiN_ is the density of TiN (5.43 g/cm^3^).

The expression of the actual contents of nitrogen at the front of the solidification interface is as follows.

$$w[N]=\frac{w{\left[N\right]}_{0}}{1‐(1‐k_{N})f_{s}}$$ (18)

where *w*[N]_0_ is the initial mass fraction of nitrogen in molten steel, and *k*_N_ is the equilibrium distribution coefficient of nitrogen in the mushy zone, which is equal to 0.40 in this work. *f*s is the solid phase fraction.

The local solidification time (*τ*) can be calculated through Eq (19).


$$\tau =\frac{T_{l}‐T_{s}}{R_{c}}$$ (19)

Here, *R*_c_ is the cooling rate.

For three target steels (Fe-0.3wt.%Ti, Fe-0.5wt.%Ti, and Fe-0.7wt.%Ti), the variables (titanium content, nitrogen content, and cooling rate) can be combined in three different groups, which is shown in Table 6.

**Table 6:** The grouping of different variables.

Group	Variables		
	Titanium content	Nitrogen content	Cooling rate
1	*w*[Ti] = 0.3 wt.%	*w*[N] = 0.0045 wt.%	288 K/min
2	*w*[Ti] = 0.5 wt.%		
3	*w*[Ti] = 0.7 wt.%		

**Fig 9:**
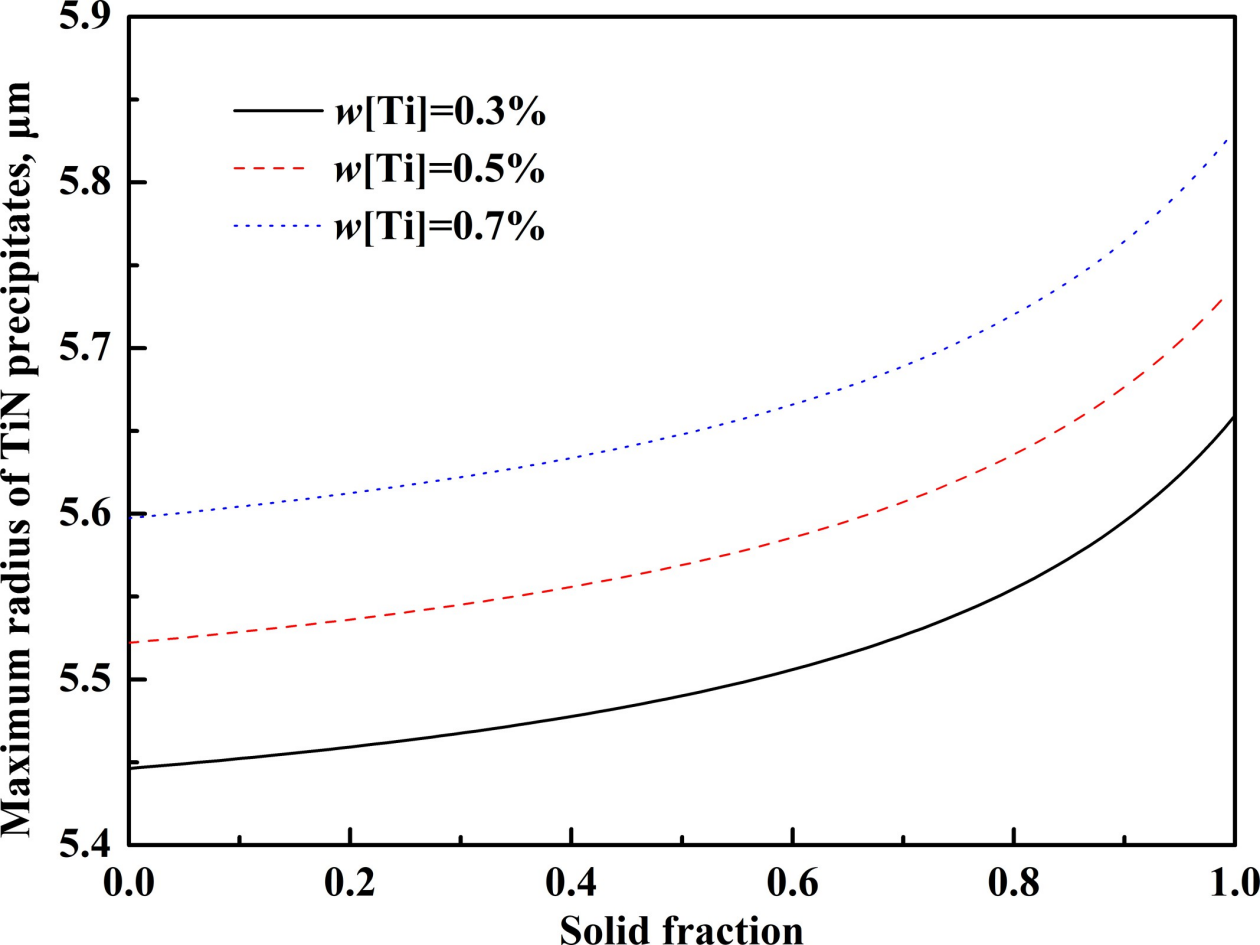
The relationship between the maximum size of precipitates and the titanium content in steel.

By substituting the data in Table 6 into the above equations, the relationship between the maximum size of precipitates and the titanium content in steel can be obtained, as shown in Fig 9.

When the cooling rate of molten steel is 288 K/min, the size of TiN precipitated from the liquid phase will gradually increase with the solidification process. With the increase of *w*[Ti], the temperature at which the liquid phase begins to precipitate TiN is increased, which will lead to more sufficient time for the growth of TiN in the liquid phase, and the final size of TiN will also become larger. The atomic mass ratio of titanium element to nitrogen element in TiN inclusions is 3.42. From the perspective of inclusion precipitation, reducing 1×10^−6^ nitrogen contents is equivalent to reducing 3.42×10^−6^ titanium contents. Therefore, in actual production, nitrogen content control in high titanium steel should be the main process objective.

To highlight the role of cooling rate, several other groups of experiments were performed in this work. For three target steels (Fe-0.5wt.%Ti), the variables (titanium content, nitrogen content, and cooling rate) can be combined in three different groups, which are shown in Table 7.

**Table 7:** The grouping of different variables.

Group	Variables		
	Titanium content	Nitrogen content	Cooling rate
4	*w*[Ti] = 0.5 wt.%	*w*[N] = 0.0045 wt.%	285 K/min
5			288 K/min
6			291 K/min
7			294 K/min

**Fig 10:**
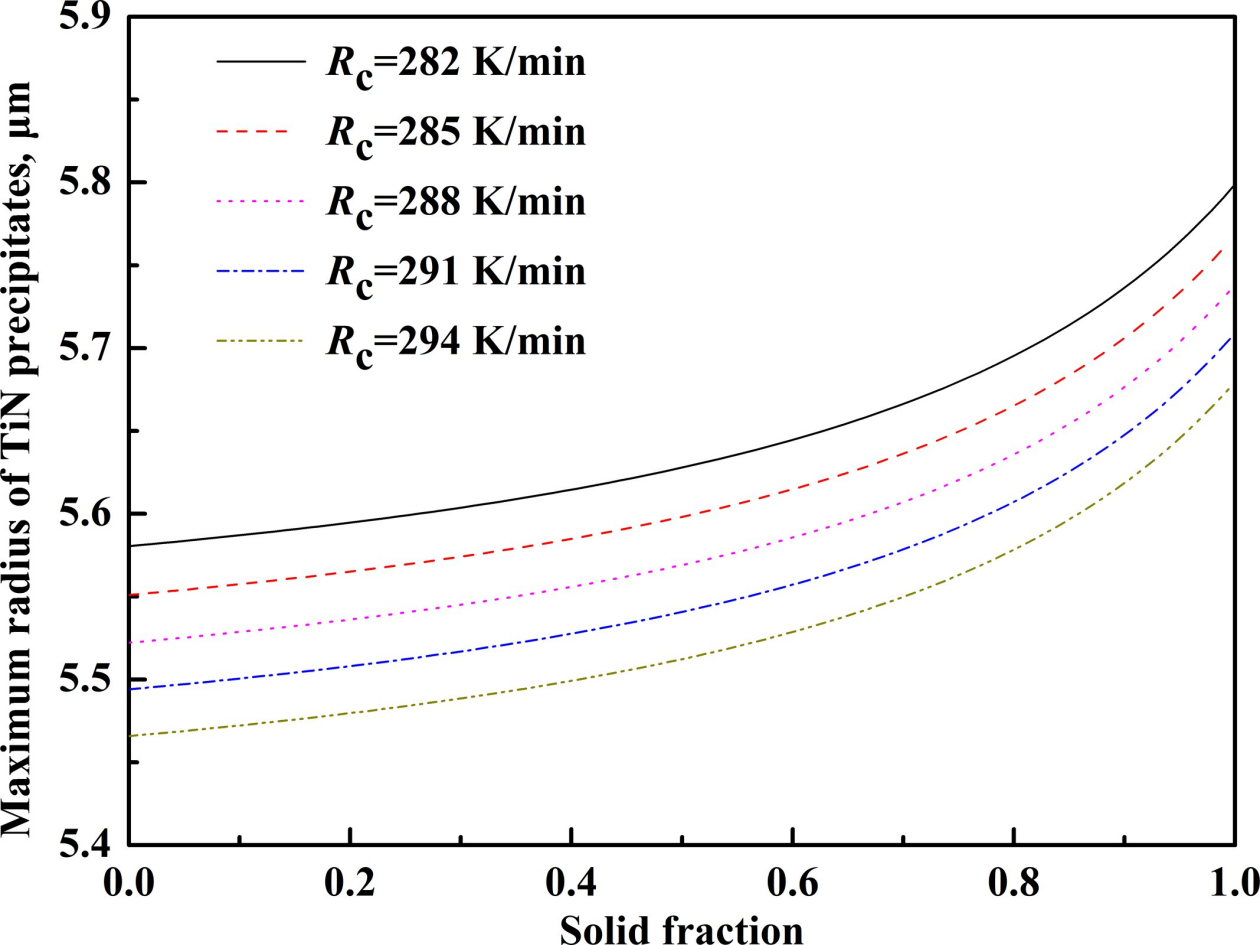
The relationship between the maximum size of precipitates and the cooling rate.

By substituting the data in Table 7 into the above equations, the relationship between the maximum size of precipitates and the cooling rate can be obtained, as shown in Fig 10.

The results show that TiN begins to precipitate in the liquid phase, but with the increase in cooling rate, the maximum size of TiN decreases gradually at the end of solidification. To achieve the effect of controlling TiN size, when the initial Ti and N contents of molten steel are constant, the determining factor affecting the maximum size of TiN will be changed to the cooling rate. In the non-equilibrium state, increasing the cooling rate will reduce the temperature at which the liquid phase begins to precipitate TiN, and the corresponding TiN growth rate at low temperature will also decrease. When the cooling rate increased from 282 K/min to 294 K/min, the maximum size of TiN at the end of solidification decreased from 5.8 μm to 5.5 μm. Therefore, in addition to effectively controlling the N content in the molten steel, the cooling rate should be appropriately increased to reduce the size of TiN in the high titanium steel, suppress the size of TiN precipitation, and reduce the harm of TiN inclusions to the steel products.

## 5 Conclusion

Through analysis of the result, the conclusions are as follows:

1. When the Ti content in the steel is 0.3 wt.%, TiN inclusions have begun to form in the liquid phase while TiC inclusions can be precipitated in the solid phase. When the Ti content increases from 0.3 wt.% to 0.5 wt.% or even 0.7 wt.%, TiN inclusions still precipitate in the liquid phase and TiC inclusions will precipitate in the mushy zone.
2. When the Ti content is more than 0.3 wt.%, TiN has been formed in the molten steel before solidification, and TiN inclusions continue to precipitate and aggregate each other with the solidification process. When the temperature is further decreased, TiC inclusions will be formed in the molten steel, and the newly precipitated inclusions of TiC and TiN will aggregate and grow together. Finally, the TiC_*x*_N_1-*x*_ inclusions will be formed at room temperature.
3. With the increase of titanium content, the number of titanium compounds precipitated in the liquid phase also increases, and the initial precipitation temperature of titanium compounds in the steel increases with the increase of titanium content. Casting temperature has little effect on the precipitation of titanium compounds in molten steel, and different Casting temperature has little effect on the initial precipitation temperature of titanium compounds.
4. Ti content and cooling rate in molten steel influence the precipitation size of TiN. With the increase of Ti content in steel, the precipitation size of TiN increases, while with the increase in cooling rate, the precipitation size of TiN decreases correspondingly.
